# A Comparison between Nanogratings-Based and Stress-Engineered Waveplates Written by Femtosecond Laser in Silica

**DOI:** 10.3390/mi11020131

**Published:** 2020-01-24

**Authors:** Jing Tian, Heng Yao, Maxime Cavillon, Enric Garcia-Caurel, Razvigor Ossikovski, Michel Stchakovsky, Celine Eypert, Bertrand Poumellec, Matthieu Lancry

**Affiliations:** 1 Institut de Chimie Moléculaire et des Matériaux d’Orsay, Université Paris Saclay, 91405 Orsay CEDEX, France; jing.tian@u-psud.fr (J.T.);; 2 LPICM, CNRS, Ecole Polytechnique, Institut Polytechnique de Paris, 91128 Palaiseau, France; 3 HORIBA Europe Research Center, 91120 Palaiseau, France

**Keywords:** femtosecond laser processing, silica glass, birefringent devices, stress birefringence

## Abstract

This paper compares anisotropic linear optical properties (linear birefringence, linear dichroism, degree of polarization) and performances (absorption coefficient, thermal stability) of two types of birefringent waveplates fabricated in silica glass by femtosecond laser direct writing. The first type of waveplate is based on birefringence induced by self-organized nanogratings imprinted in the glass. One the other hand, the second design is based on birefringence originating from the stress-field formed around the aforementioned nanogratings. In addition to the provided comparison, the manufacturing of stress-engineered half waveplates in the UV-Visible range, and with mm-size clear aperture and negligible excess losses, is reported. Such results contrast with waveplates made of nanogratings, as the later exhibit significantly higher scattering losses and depolarization effects in the UV-Visible range.

## 1. Introduction

In 2003, a new type of self-organized structures was observed inside SiO_2_ glass after irradiation with an ultrafast femtosecond laser [1]. Such structuration of the glass was found to be strongly anisotropic [2,3,4]. These highly ordered sub-wavelength structures with lamellae-like oxygen-deficient regions are oriented perpendicular to the incident beam polarization [1,5]. A decade ago, Bricchi et al. demonstrated that such thermally stable nanostructures [6], due to their sub-wavelength periodicity, behave as a negative uniaxial birefringent material where the fast axis (slow axis) is parallel (perpendicular) to the orientation of the laser polarization [7]. These induced birefringent modifications are ideal candidates to design numerous optical elements such as Fresnel zone plates, lens-based spin filters, polarization gratings, radial/azimuth polarization converters, Airy beam converter and high-order laser mode converters [8,9,10,11]. Additionally, multi-dimensional optical data storage with unprecedented thermal stability has been demonstrated [12,13], as well as micro-waveplates and their arrays [14,15], or achromatic polarization convertor [16] for polarimetry applications.

Although of considerable interest, birefringent optics made of nanogratings exhibit significant drawbacks. One of them is the particularly high photo-induced losses in both visible (Vis) and ultraviolet (UV) regions [17]. This is caused by the intrinsic nanoporous nature [18] of the nanogratings yielding a significant amount of Rayleigh scattering. This drawback also couples with significant light depolarization [19], which is detrimental for most applications. Additionally, the effect of form birefringence, unlike intrinsic birefringence due to the anisotropy of oriented molecules, is induced by the alignment of submicroscopic rodlets or platelets. In our case nanogratings are made of isotropic objects (the nanopores) organized in an anisotropic way (in nanoplanes). This results into a strong birefringence response for wavelengths longer than the nanogratings periodicity (λ >> Λ). On the other hand, for λ ≈ L, this response is strongly decreased to a level that is no more exploitable for most applications.

Moreover, formation of nanogratings is not solely creating form birefringence but also a stress-birefringence contribution, attributed to glass quenching and morphological changes that participate to the total birefringence [20,21,22]. Indeed, irradiation of silica glass by a femtosecond laser beam leads to a net volume expansion [23,24], which correlates with the formation of porous structures inside the nanogratings [18,25]. The effective glass volume is reduced, which correlates to the appearance of a permanent strain. The later results into elastic strain as a direct response of the material, and therefore into stress within and around the laser-modified region. By cleaving the laser-modified samples, part of the elastic strain relaxes, and a valley-like surface topography indicates the occurrence of glass densification. Several research groups have shown the presence of a zone of mechanical stresses, possibly associated with birefringence properties in the irradiated zone, related to the polarization of the incident laser beam [26]. Depending on the laser exposure conditions, the overall stress can be enhanced or minimized [24] leading to tunable birefringence values from 10^−5^ up to ~10^−3^ [27,28,29]. Such stress-induced birefringence influences the inscribed object optical properties, and can lead to the formation of undesired cracks, especially in a multilayer structure made of subsequent irradiations at different depth levels. Therefore, it results in a complicated and unreliable writing procedure where the stress must either be considered or eliminated.

Alternative approaches are based on stress-induced birefringence resulting from different conditions such as process-induced temperature gradient, applied external pressure (from mounting hardware, etc.), or again primary manufacturing processes (thermal expansion coefficient mismatch in optical fibers manufacturing, etc.). For example, recent research has been conducted to use stress-induced birefringence in a glass plate to generate an optical vortex and full Poincare beams (optical beams that have every possible polarization states across its cross-section) [30]. Within last years there are few examples of stress-engineered optical elements made by femtosecond laser direct writing (FLDW) for waveguides applications. For example, the Herman group has developed the fabrication of integrated optical components for polarization control, like guided wave retarders and polarization beam splitters [28,31,32]. In this work, the possibility of tuning the waveguide birefringence in fused silica was explored by inducing stressors with femtosecond written laser tracks formed parallel to the waveguides. By exploiting this stress, together with the form birefringence generated by the laser-formed nanogratings, the prospects of either increasing or decreasing the waveguide birefringence is allowed.

In addition, this opens the door towards the design of polarization-dependent devices not only in waveguides but also for free space optics as recently demonstrated by Y. Bellouard’s group. Unlike fs-laser nanogratings or laser-oriented liquid crystal devices, this provides a “birefringent clear aperture” that is free from laser direct modifications. Within last years micro-lens formation was reported [33], along with the formation of 1 mm clear aperture waveplate with retardance up to 50 nm [34], i.e., a quarter waveplate at 200 nm. Very recently, optical components exploiting Pancharatnam-Berry phase were implemented by harnessing femtosecond laser based stress–induced birefringence. For example, beam converters to obtain beams with orbital angular momentum were demonstrated in glass and crystalline materials using circular polarized light to imprint “stressors” and resulting space variant birefringence [35].

In this context, the goal of this paper is to compare the anisotropic linear optical properties of a waveplate made of nanogratings with a clear aperture waveplate based on stress-induced birefringence. Therefore, Mueller-matrix spectroscopic ellipsometry is used as it provides the spectral dispersion of linear birefringence *LB*, linear dichroism *LD*, *LB* neutral axis orientation and the depolarization rate from the UV to the Near-IR range. Moreover, a comparison of both optical losses and thermal stability between these two alternative ways will be provided as well, enabling engineering of uniform and space-selective birefringent optical components.

## 2. Materials and Methods

The initial principle of femtosecond (fs) laser imprinted stress-induced waveplates has been introduced and described in detail in reference [34]. In the present case, a square shaped clear aperture was defined by writing two sets of lines (each single line is called a stressor) in a multilayer approach called ‘stressor bars’ throughout the paper. The laser beam was produced by a femtosecond laser system operating at λ = 1030 nm and delivering 250 fs pulses at a repetition rate of 100 kHz with a typical average power up to 10 W (Amplitude Systèmes, Pessac, France). The beam was focused to different depths below the front face of 3 mm thick silica glass plates (SuprasilCG, Heraeus, Hanau, Germany) using a 0.16 NA aspheric lens (estimated beam waist *w* ~3.5 µm). Based on preliminary experiments the laser energy and the scanning speed were chosen so that the irradiated region falls within the type II regime (type 0.4 µJ/pulse, 1 mm/s speed in our conditions) corresponding to the formation of nanogratings in silica glass.

**Figure 1:**
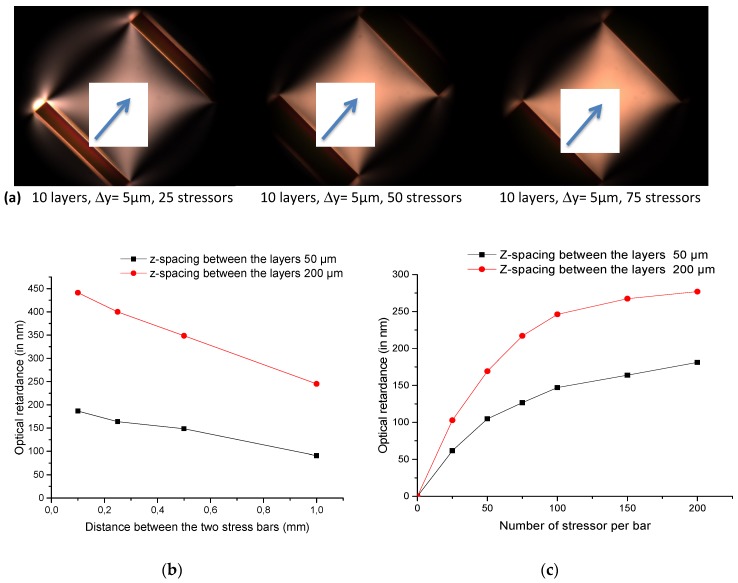
(**a**) Typical optical microscope images of stress-engineering waveplates taken between in crossed polarizers. Blue arrow indicates the center of the clear aperture. (**b**) Optical retardance *R* (in nm) as a function of the distance *d* (in mm) between the two stress bars (stressor gap 5 µm, 100 stressors per bar, 10 layers). (**c**) Optical retardance *R* measured at 550 nm as a function of the number of stressors per bar. The clear aperture was fixed to 1 mm. Conditions: SuprasilCG glass, λ = 1030 nm; 250 fs; 100 kHz; 1 mm/s scanning speed; numerical aperture 0.16 NA; 2 µJ/pulse.

It has been reported by Bellouard’s group that the stress distribution around the laser-exposed area depends on the laser polarization (as it controls the nanogratings orientation) [26]. Therefore, here we chose to investigate a specific writing configuration that maximizes the stress amplitude around a laser track as well as minimizes the imprinting of anisotropic circular optical properties (within the laser affected zone) such as circular dichroism and circular birefringence [36,37]. The glass sample was moved along an axis defined as the Y-axis (or scanning direction). The laser linear polarization was oriented perpendicular to such axis (along the x-axis). This writing configuration is defined as “Yx” configuration of writing. The laser-induced nanogratings wave-vector is thus oriented perpendicularly to the laser scanning direction. In this simple arrangement, we did not use the “etched cuts” as initially proposed by McMillen et al. [34]. However, the stress-induced birefringence remains confined within the area of interest as visible in Figure 1a.

Optical retardance of the laser-induced modifications, defined as the product of linear birefringence (*LB*) by the thickness of the birefringence object (*l*), i.e., *R* = *LB* × *l*, is measured using an Olympus BX51 polarizing optical microscope (Olympus, Tokyo, Japan) equipped with a “de Sénarmont” compensator. The “de Sénarmont” compensator couples a high precision quarter wave birefringent plate with a 180-degree rotating analyzer to provide retardation measurements in the visible range i.e., at 550 nm in the present paper. Such setup has an accuracy that approaches a few nm when used in our conditions. Additionally, UV-Vis-NIR absorption spectra were performed using a Cary 5000 spectrophotometer (Agilent, Santa Clara, California, USA) with a data interval of 0.5 nm. Anisotropic optical properties were investigated using a phase modulated spectroscopic ellipsometer (UVISEL+, HORIBA Scientific, Kyoto, Japan) over λ = 200 to 1500 nm spectral range. Since this equipment gives access only to the three first columns of the Mueller matrix [38], we used a recent approach [39,40] allowing for the completion of an experimental nondepolarizing Mueller matrix with a column or a row missing to a full, 16-element one. All measurements were made using a collimated probe beam in normal incidence. The probe beam size of these two instruments was fixed to 0.8 mm for all measurements. The samples were oriented in such a way that their writing/scanning axis was set horizontal +/−1° in the reference frame of the Mueller ellipsometer.

## 3. Results

As a preliminary work, based on the results published by McMillen et al. [34], the design has been tailored so that we could reach a high optical retardance *R* of 200 nm (i.e., a half waveplate at 400 nm) or even more, with a 1 mm × 1 mm clear aperture, through careful control of the number, density, layers and laser exposure parameters of the stressors. So, in the following, we created several twin columns of lines (defined as “stress bar” above) with a typical spacing *d* ranging from 0.1 mm up to 1 mm, which defines the clear aperture. Each stress bar is made up of an assembly of 10 layers written with a spacing *Δz* of either 50 µm or 200 µm. The pulse energy was fixed to 2 µJ/pulse for the numerical aperture used in this study namely a 0.16 NA aspheric lens. Some typical polarized optical microscope images of three stress-engineered waveplates are shown in Figure 1a. These images were obtained in transmission mode with the sample oriented at 45° between crossed polarizers.

In the first set of results presented in Figure 1b, the parameter of the study is the distance, *d,* between the stress bars that defines the clear aperture of the stress-engineered waveplate. The fixed parameters were as follows: a stressor gap *Δy* = 5 µm, 100 stressors per bar and 10 layers per stress bar with a *Δz* spacing of either 50 or 200 µm. As it can be seen in Figure 1a, there is a significant decrease in the photo-induced retardance *R* when the clear aperture size *d* is increased. In addition, increasing the layer spacing *Δz* allows the writing of a much higher retardance, in agreement with the use of a low NA, which leads to the imprinting of quite thick laser tracks in the laser propagation direction.

In the second set of experiments, displayed in Figure 1c, we changed the number of lines for a relatively large clear aperture of 1 mm, which allows one to consider the development of birefringent optics from this design. In this figure, the retardance values measured at the center between the two stress bars increase when the number of stressors increases. There is also a tendency for the retardance value to saturate when the number of stressors is greater than 100. The amplitude of the retardance reaches almost 180 nm for *Δz* = 50 µm layer spacing and 280 nm for *Δz* = 200 µm, which makes possible envisioning the production of both half and quarter waveplates in this configuration.

As a first approximation, the Mueller matrix of the “stress-engineered waveplate” can be approximated by the Mueller matrix of a perfect linear retarder. In contrast, the waveplate made of nanogratings exhibits some significant linear dichroism especially in the UV-Vis range [17,19] but also some anisotropic circular properties depending on the writing configuration [36,37]. Then, by using the differential decomposition described in [41,42], it is possible to extract all the polarimetric properties from the Mueller matrix of a sample provided that the laser track is considered as homogeneous in the direction of light propagation. As described in Ref. [41] the polarimetric optical response of a medium of length *l*, with a complex refractive index $\tilde{n}=n+i\cdot \kappa$, can be defined as a superposition of the following basic polarimetric properties: linear birefringence $LB=\frac{2\pi }{\lambda }\cdot \left(n_{X}-n_{Y}\right)\cdot l$, 45° linear birefringence $LB^{\prime }=\frac{2\pi }{\lambda }\cdot \left(n_{45}-n_{135}\right)\cdot l$, linear dichroism $LD=\frac{2\pi }{\lambda }\cdot \left(\kappa_{X}-\kappa_{Y}\right)\cdot l$, 45° linear dichroism $LD^{\prime }=\frac{2\pi }{\lambda }\cdot \left(\kappa_{45}-\kappa_{135}\right)\cdot l$, circular birefringence $CB=\frac{2\pi }{\lambda }\cdot \left(n_{L}-n_{R}\right)\cdot l$, circular dichroism $CD=\frac{2\pi }{\lambda }\cdot \left(\kappa_{L}-\kappa_{R}\right)\cdot l$, together with the Degree of Polarization (termed *DoP*).

**Figure 2:**
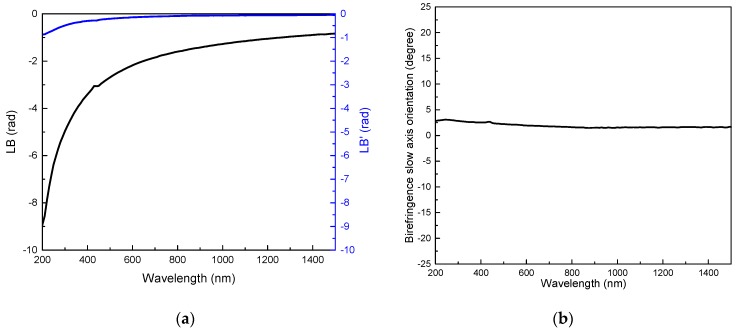
Experimental values of effective linear anisotropic optical properties for 1 mm clear aperture stress waveplate: (**a**) Linear birefringences LB and LB’ (defined in text). (**b**) Slow axis orientation (in degree) of the linear birefringence as a function of the wavelength. Waveplate design: stressor gap *Δy* = 5 µm, 100 stressors per bar, 10 layers with *Δz* = 50 µm.

According to the data shown in Figure 2a, we can observe a strong negative *LB* whose amplitude (in radians) is monotonously increasing at low wavelengths and reaches −π rad around 450 nm (i.e., a half waveplate at this wavelength). It can also be seen that the *xy* linear birefringence *LB* is one order of magnitude higher than the 45°-birefringence (*LB’*). From these two curves, the azimuthal *θ_LB_* of the birefringence orientation (*θ_LB_* = 0.5.atan (*LB’/LB*)) is calculated, and is displayed in the Figure 2b. Such calculations reveal that the stress-induced birefringence exhibits a slow axis orientation more or less parallel (±1–2°) to the reference x-axis. This confirms that the arrangement of opposing stress bars has induced a quasi-uniaxial loading of the material in the center of the clear aperture, in addition to creating a strong optical retardance. This agrees with the design where nanogratings (wave-vector) are oriented perpendicularly to the laser scanning direction Y, such that the principal component of the stress tensor [26,43] is directed perpendicular to the lines/stressors orientation. As for the anisotropic circular optical properties, we did not observe any significant *CD* or *CB* in the investigated spectral range.

**Figure 3:**
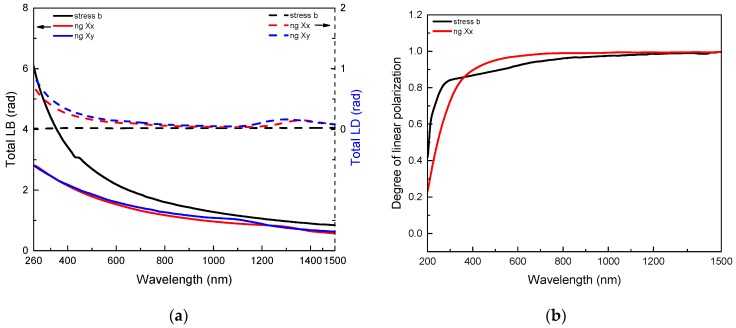
(**a**) Spectral dependence of the anisotropic linear optical properties *Total LB* (continuous lines) and *Total LD* (dashed lines) extracted from Mueller matrix decomposition. Black lines are for the stress-engineered waveplate (a half waveplate at 450 nm) whereas red and blue lines are for nanogratings-based waveplates written in Xx and Xy configurations. (**b**) Spectral dependence of the linear Degree of Polarization.

Figure 3a shows “*Total LB*” and the “*Total LD*” corresponding to the following equations: $Total\;LB=\sqrt{LB^{2}+LB^{{}^{\prime }2}}$ and $Total\;LD=\sqrt{LD^{2}+LD^{{}^{\prime }2}}$. Note that these two properties are thus independent of the azimuthal orientation of the samples with respect to the polarimeter reference frame. Apart from the weak dichroic band around 1200–1400 nm which has been discussed in a previous publication [19], the anisotropic linear optical properties of the nanogratings waveplates (type a quarter waveplate at 500 nm) are mostly attributed to the formation of sub-wavelength nanolayers resulting in a strong form birefringence. However, there is also a contribution of stress-induced birefringence, which has been partly investigated in the literature [23,24,26,44]. We reported earlier that the spectral dependence of the optical path length difference expressed as $\left(n_{X}-n_{Y}\right)\cdot l$ is quite flat in the Vis and Near-IR spectral range, which results in an increase of $LB=\frac{2\pi }{\lambda }\cdot \left(n_{X}-n_{Y}\right)\cdot l$ with decreasing the probe wavelength, as it can be seen here. However, the measured LB dependence with the wavelength shows a steady decrease in the spectral region from 200 to 300 nm for Xx writing configuration and 200–400 nm for Xy writing configuration. This trend is expected by the effective medium theory as, at short wavelengths, the nanogratings period Λ approaches the probe light wavelength λ. In contrast, the TLB of the stress-engineered waveplate (a half waveplate at 450 nm) still follows a monotonous increase for decreasing wavelengths down to 200 nm, which allows, in principle, to imprint waveplates in the UV range.

An increase of *Total LD* at short wavelengths accompanies the linear birefringence in the nanogratings regime as observed in Figure 3a. The positive *LD* (and nearly zero *LD’* not shown here) implies that higher losses were measured for polarization oriented perpendicular to the nanolayers in agreement with [17]. It is known that a layered medium, made of alternating layers of two different isotropic materials with complex refractive indices, exhibits a linear dichroism [45]. Note that the linear dichroism observed in the UV-Vis range should be rather called linear diattenuation since it can be mostly attributed to polarization dependent scattering [2,17] due to the intrinsic nanoporous nature of the nanogratings [18] rather than to polarization dependent absorption. In contrast, we did not observe any linear dichroism for the stress-engineered waveplates (type *Total LD* < 0.02 rad).

Another interesting feature is the Degree of Polarization *DoP*, which is shown in Figure 3b. For λ > 600 nm all depolarization effects remain smaller than 10% and monotonically decrease with λ reaching a level below 5% for λ > 800 nm. However, the *DoP* strongly decreases in the UV range reaching less than 80% below 250 nm. In the case of the stress-engineered waveplate depolarization is likely due to the fact that the distribution of the polarimetric properties (birefringence and dichroism) is not homogeneous within the area probed by the light beam i.e., the clear aperture (see Figure 1a). In consequence there is a non-coherent addition of contributions with different polarization states at the level of the detector, which creates the measured depolarization. However the in the case of the nanogratings-based waveplate, the observed depolarization effects are more likely due to presence of nanopores that generates some strong scattering effects in the UV-Vis range. The latter create a random distribution of polarization states which incoherently add at the level of the detector, and which can be properly described in the frame of the randomly fluctuating media approximation by Ossikovsky and Arteaga [42].

In terms of optical performance, the transmission spectra (200–1700 nm) of the laser written waveplates were measured using a spectrometer (Cary 5000). To this end, the nanogratings samples were first annealed at 600 °C for 2 hours to bleach absorption bands observed at the short wavelengths, which are attributed to SiE’ centers at 210 nm (i.e., ≡Si, corresponding to an unpaired electron in a silicon atom bound to three oxygen atoms), and oxygen deficiency center (ODC)(II) at 245 nm, (–O–Si–O–, a divalent silicon atom) [46]. The erasure of these defects resulted in significantly lower losses from 200 to 500 nm, as published earlier [17,47,48] without significantly affecting the linear birefringence.

**Figure 4:**
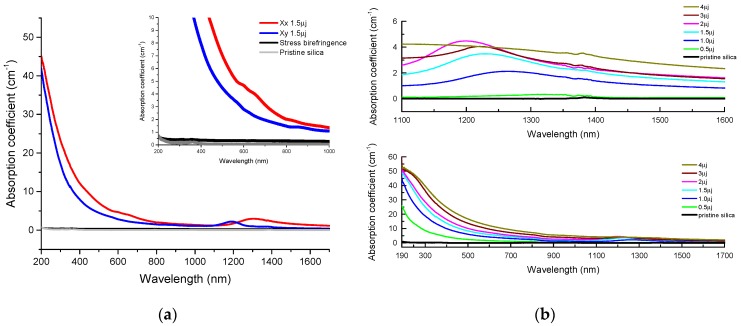
(**a**) Comparison of absorption coefficient *k_abs_*(*λ*) spectra for different waveplates together with pristine silica. Black line is for the stress-engineered waveplate (a half waveplate at 450 nm) whereas red and blue lines are for nanogratings-based waveplates written in Xx and Xy configurations. (**b**) Absorption coefficient *k_abs_*(*λ*) spectra of nanogratings-based waveplates for different pulse energies. Note that each waveplate was annealed for 2 hours at 600 °C prior to measurements.

Following this, the transmission spectra were corrected by removing the multiple-reflection spectral losses that are independent of the sample thickness and we calculated the internal transmittance *T_int_λ*) as well as the absorption constant *k_abs_*(*λ*) expressed in cm^−1^. Figure 4a shows the absorption spectra of the stress-engineered waveplate compared both to the nanogratings-based waveplate and the pristine silica substrate. Figure 4b exhibits the dependence of spectra with the pulse energy within the nanogratings regime. The main contribution to the losses observed for the nanogratings-based waveplate originates from the Rayleigh scattering of the inhomogeneous structure, which has dependence in 1/λ^4^ leading to strong losses in the UV-Vis range. In addition, there is a weak absorption band in the 1200–1400 nm spectral range, which was discussed in [19] in the form of a strong linear dichroism. However, the attribution of this band is still not clear. Comparison to the absorption coefficient for the stress-engineered waveplate reveals negligible absorption much below 1 cm^−1^ over the entire spectral range, i.e., of same order of magnitude as that of the pristine sample. This experience clearly shows that while the nanogratings create a strong scattering effect in the UV range, which is detrimental to the optical performance of the device, the stress-engineered sample does not show this drawback, which is an important point in favor of the latter technologies when it comes to use short wavelengths.

**Figure 5:**
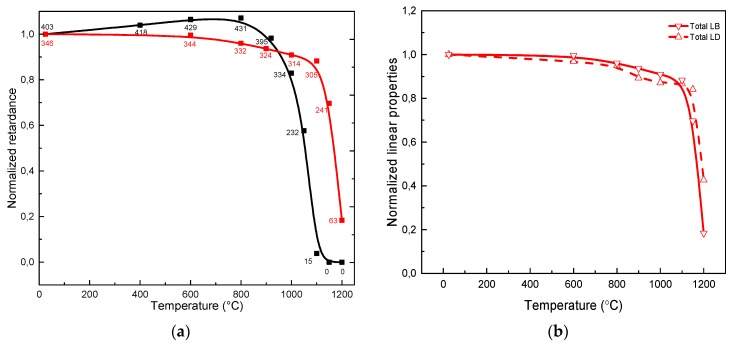
(**a**) Experimental values of the normalized evolution of optical retardance *R* (proportional to *LB*) at 550 nm with annealing temperature. Note we add the retardance values for each point as a label. Black dots are for the stress-engineered waveplate whereas red squares are for a nanogratings-based waveplate written in Xy configuration. (**b**) Normalized evolutions of *Total LB* and *Total LD* according to annealing temperature. Lines are guides to the eye.

An advantage giving rise to a significant interest in nanogratings and related birefringent optical components is the so-called “extraordinary” thermal stability reported in 2006 [6]. This has led to the development of 5D optical data storage with seemly-unlimited lifetime by Kazansky’s group [13], as well as to the extensive studies of Fiber Bragg Gratings for structural health monitoring in high temperature environment, which has triggered the fabrication of commercial fiber sensors based on nanogratings (FemtoFiber Tech@, Berlin, Germany; FemtoSensing@, Atlanta, GA, USA). Consequently, in Figure 5a is displayed a comparison of the thermal stability of the waveplates, studied through an annealing experiment of isochronal (*Δt* = 30 min) annealing steps (*ΔT* = 50 °C). It is worth pointing out that the curves in Figure 5 represents the “stability curve” provided that the criterion (*δt*k_0_)^-(ΔT/Tmax)^ ≪ 1 is fulfilled [49] and where *k*_0_ is the pre-exponential factor in the Arrhenius rate constant of the erasure reaction. For nanogratings written in silica, *k*_0_ has been estimated to be around de 5.10^5^–5.10^7^ s^−1^ [50] depending on the laser writing parameters. Therefore, when this criterion is respected, each point can be considered independent to each other.

In agreement with the literature, the nanogratings-based waveplates (both *Total LB* and *Total LD* as shown in Figure 5b) can survive hours to temperatures higher than 1100 °C in SiO_2_ and typically erase for annealing treatments around the glass annealing temperature *T_a_* (i.e., 1120 °C for SuprasilCG and with *T_a_* defined as *ŋ*(*T*) = 10^13^ dPa.s^−1^). In contrast, we can observe the reduction of the stress-induced birefringence by a factor of two after 30 min of annealing at 1050 °C. This agrees with the expanded idea that an annealing at around 0.8–0.9.*T_a_* (depending on the duration of the thermal treatment) should relax the stress-induced birefringence, as it is well known in glass manufacturing. Following this view, A. Čerkauskaitė [50] has demonstrated that annealing of nanogratings-based samples for 24 hours at 950 °C, 6 hours at 1000 °C, 2 hours at 1050 °C, and 1 hour at 1100–1150 °C “totally” eliminates the stress-induced birefringence. In contrast, since the annealing of nanogratings is governed by the slow decay term, the form birefringence remains after these thermal treatments.

## 4. Conclusions

In conclusion, spectral properties of stress-engineered waveplates made by femtosecond laser direct writing were characterized for a wide range of wavelengths from 200 nm to 1500 nm. The transmission spectra of stress-engineered waveplates show that such objects exhibit only minor increment of the absorption constant with respect to the silica substrate, and it remains below 1 cm^−1^ in the investigated spectral range. In contrast, nanogratings-based waveplates present strong losses accompanied with a significant linear dichroism, which are attributed to the scattering induced by the intrinsically-nanoporous layers constituting the fabricated waveplate.

The spectral dispersion of anisotropic optical properties was reported, demonstrating the possibility to imprint stress-engineered half-waveplates in the UV-Vis range with no linear dichroism and minor optical losses. In addition, these stress-induced birefringent waveplates were stable up to 1000 °C, making them attractive candidates for UV-Vis birefringent and space variant birefringent devices.
